# A more randomly organized grey matter network is associated with deteriorating language and global cognition in individuals with subjective cognitive decline

**DOI:** 10.1002/hbm.24065

**Published:** 2018-03-30

**Authors:** Sander C. J. Verfaillie, Rosalinde E. R. Slot, Ellen Dicks, Niels D. Prins, Jozefien M. Overbeek, Charlotte E. Teunissen, Philip Scheltens, Frederik Barkhof, Wiesje M. van der Flier, Betty M. Tijms

**Affiliations:** ^1^ Department of Neurology and Alzheimer Center VU University Medical Center, Amsterdam The Netherlands; ^2^ Department of Clinical Chemistry VU University Medical Center Amsterdam The Netherlands; ^3^ Department of Radiology & Nuclear Medicine VU University Medical Center, Amsterdam The Netherlands; ^4^ Institutes of Neurology and Healthcare Engineering UCL London United Kingdom; ^5^ Department of Epidemiology & Biostatistics VU University Medical Center, Amsterdam The Netherlands

**Keywords:** Alzheimer's disease, cognition, connectivity, graph theory, grey matter network, longitudinal, mild cognitive impairment, MRI, subjective cognitive decline

## Abstract

**Objectives:**

Grey matter network disruptions in Alzheimer's disease (AD) are associated with worse cognitive impairment cross‐sectionally. Our aim was to investigate whether indications of a more random network organization are associated with longitudinal decline in specific cognitive functions in individuals with subjective cognitive decline (SCD).

**Experimental design**: We included 231 individuals with SCD who had annually repeated neuropsychological assessment (3 ± 1 years; *n* = 646 neuropsychological investigations) available from the Amsterdam Dementia Cohort (54% male, age: 63 ± 9, MMSE: 28 ± 2). Single‐subject grey matter networks were extracted from baseline 3D‐T1 MRI scans and we computed basic network (size, degree, connectivity density) and higher‐order (path length, clustering, betweenness centrality, normalized path length [lambda] and normalized clustering [gamma]) parameters at whole brain and/or regional levels. We tested associations of network parameters with baseline and annual cognition (memory, attention, executive functioning, language composite scores, and global cognition [all domains with MMSE]) using linear mixed models, adjusted for age, sex, education, scanner and total gray matter volume.

**Principal observations**: Lower network size was associated with steeper decline in language (β ± SE = 0.12 ± 0.05, *p* < 0.05FDR). Higher‐order network parameters showed no cross‐sectional associations. Lower gamma and lambda values were associated with steeper decline in global cognition (gamma: β ± SE = 0.06 ± 0.02); lambda: β ± SE = 0.06 ± 0.02), language (gamma: β ± SE = 0.11 ± 0.04; lambda: β ± SE = 0.12 ± 0.05; all *p* < 0.05FDR). Lower path length values in precuneus and fronto‐temporo‐occipital cortices were associated with a steeper decline in global cognition.

**Conclusions:**

A more randomly organized grey matter network was associated with a steeper decline of cognitive functioning, possibly indicating the start of cognitive impairment.

## 1 INTRODUCTION

The pathogenesis of Alzheimer's Disease (AD) takes years, eventually causing progressive neurodegeneration and cognitive decline (Jack et al., 2013; Scheltens et al., 2016). Self‐reported subjective cognitive decline (SCD) in cognitively intact individuals might be one of the first symptoms of AD (Jessen et al., 2014, 2010; Schmand, Jonker, Hooijer, & Lindeboom, 1996). About 25–50% of people older than 60 years perceive cognitive decline (Jonker, Geerlings, & Schmand, 2000), but longitudinal studies show that only a minority (11–16%) of individuals with SCD shows clinical progression over time (Jessen et al., 2010; Van Harten et al., 2013). At this point it remains difficult to understand which individuals with SCD will show future cognitive decline.

Possibly, structural magnetic resonance imaging (MRI) might help identifying those at risk of decline (Dickerson et al., 2009; Tijms et al., 2016; Verfaillie et al., 2016). Evidence is accumulating that brain changes leading to cognitive decline and dementia are not restricted to specific regions such as the medial temporal lobe, but rather include widespread changes in structure, function and organization of the brain (Benzinger et al., 2013; Dickerson et al., 2009; Pegueroles et al., 2017). Patterns of grey matter morphology can be described as a network consisting of multiple small regions of grey matter (i.e., nodes) that are connected to each other when they show structural similarity within a cortex across subjects. The advantage of a network representation of grey matter morphology is that it provides an opportunity to precisely quantify individual brains with tools from graph theory. For example, the small world coefficient indicates whether the organization of connections within a network is different compared to those of a randomly organized network. Although, the biological meaning of structural similarities is not yet fully understood, grey matter similarity has been demonstrated to be associated with synchronized maturation between brain regions (Alexander‐Bloch and Bullmore, 2013; Alexander‐Bloch, Raznahan, Bullmore, & Giedd, 2013b; Andrews, Halpern, & Purves, 1997), which might be reflected by a higher degree of clustering. Previous cross‐sectional studies have demonstrated that alterations of grey matter network parameters are associated with disease severity (Pereira et al., 2016; Tijms et al., 2013a; Yao et al., 2010; Zhou and Lui, 2013) and with the degree of cognitive impairment in AD (Tijms et al., 2014). A group‐based network study suggested that grey matter networks that more resemble randomly organized networks are associated with future progression to dementia in individuals with mild cognitive impairment (Pereira et al., 2016). Grey matter networks may start to become more randomly organized in early, preclinical stages of the disease (Tijms et al., 2016), and in particular lower values of the clustering coefficient seem to be associated with faster clinical progression in AD (Tijms et al., 2017). Possibly, lower clustering coefficient values may reflect that AD pathological hallmarks, amyloid and tau aggregation, starts in specific brain areas, which could lead to an asynchronous loss of grey matter network organization during the development of AD. As such, it can be hypothesized that lower clustering coefficients values in grey matter networks might provide a biological explanation for cognitive decline.

The aim of the present study was to investigate whether grey matter networks parameters in individuals with SCD are related to decline in global cognition, memory, attention, language and executive functioning over time. We expected that a more random network organization as reflected by lower network values such as normalized clustering (i.e., gamma), would be related to faster cognitive decline.

## 2 METHODS

### 2.1 Study population

Two hundred thirty‐one individuals with SCD were included with available MRI and follow‐up neuropsychological assessment from the Amsterdam Dementia Cohort (Van Der Flier et al., 2014). Patients visited our memory clinic between 2000 and 2012 and were described in earlier studies (Benedictus et al., 2015; Verfaillie et al., 2017, 2016). At baseline, all patients underwent standardized dementia screening, including medical history, extensive neuropsychological assessment, physical examination, blood tests, and 3D‐T1‐weighted structural MRI (brain). Clinical diagnosis was established by multidisciplinary consensus. Subjects were labeled as having SCD (Jessen et al., 2014) when they presented with cognitive complaints, and results of clinical and neuropsychological assessments were within normal range, and criteria for mild cognitive impairment (MCI), dementia, or other disorders known to cause cognitive complaints were not met (i.e., cognitively intact). In addition, we offered patients a choice to undergo a lumbar puncture for research purposes. Cerebrospinal fluid (CSF) β‐amyloid1–42 (cutoff: <640 ng/L) and total tau (cutoff: >375 ng L^−1^) was determined using sandwich enzyme‐linked immunoassays (Innogenetics, Belgium) (Mulder et al., 2010; Zwan et al., 2016). Follow‐up visits took place annually (approximately) during which medical examination and neuropsychological assessment were repeated. The medical ethics committee of the VU University Medical Center approved the study. All patients provided written informed consent for their clinical data to be used for research purposes.

### 2.2 Neuropsychological assessment

Our neuropsychological test battery included tests that measure cognitive functioning in the domains of memory, attention, executive functioning, and language (Van Der Flier et al., 2014). For the attention domain, we used the digit span forward, Trail making Test (TMT)‐A, and Stroop1&2. For the memory domain, we used the Dutch version of the Rey auditory verbal learning test (RAVLT) direct and delayed recall, and visual association task (VAT)‐A. For the language domain, the following tests were used: Category fluency animals and VAT naming. For the executive function domain, we used: TMT‐B, digit span backwards, and Stroop color‐word test. To assess global cognitive functioning all previously mentioned tests were combined with the mini‐mental state examination (MMSE). All neuropsychological test scores were Z‐transformed using the corresponding baseline distribution as a reference. TMT‐A, TMT‐B and Stroop were inverted such that lower scores reflect worse performance. Missing data per test ranged from 1 to 10% in the longitudinal data set, missing data of each individual neuropsychological test can be found in Table 1. To avoid bias, we estimated missing values using multivariate imputation, including age, sex, education, time and all available neuropsychological test results in the model (Buuren and Groothuis‐Oudshoorn, 2011; Donders, van der Heijden, Stijnen, & Moons, 2006). Because multiple imputation relies on stochastic processes, we repeated this process fifteen times to ensure stability of the results. Subsequently, for each imputed dataset, we created composite domain scores by taking the average *Z* score of each test per domain.

**Table 1:** Baseline demographical, clinical, neuropsychological, and imaging data

Demographics	Total group*(n = 231)*
Male/female (*n*)	126/105
Age (years)	62.95 (9.22)
Education (range: 1–7)	5.31 (1.36)
Scanner type (1T/3T)	124/107
**Clinical**	
Baseline self‐reported cognitive complaints (years)	3.10 (3.62)
MMSE (*n*[%] missing: 7[1%])	28.35 (1.56)
Follow‐up time	2.80 (1.01)
β‐amyloid 1–42^1^	834.61 (265.80)
*n* < 640 pg mL^−1^ (%)	*n* = 40 (25%)
Tau (total)^1^	294.27 (179.57)
*n* > 375 pg mL^−1^ (%)	*n* = 30 (19%)
**Final follow‐up diagnosis**	
SCD *n* (%)	195 (84%)
MCI *n* (%)	28 (12%)
AD dementia *n* (%)	4 (2%)
FTD *n* (%)	2 (1%)
VaD *n* (%)	2 (1%)
**Neuropsychological Assessment (in total *n* = 646 available)**	
*Attention*	
Digit span forward (*n*[% of total]missing: 5[1%])	12.58 (3.17)
Trailmaking test A (*n*[%] missing: 13[2%])	39.81 (15.66)
Stroop word (*n*[%] missing: 66[10%])	46.31 (9.29)
Stroop color (*n*[%] missing: 66[10%])	62.53 (11.97)
*Executive function*	
Digit span backward (*n*[%] missing: 5[1%])	9.25 (2.76)
Trailmaking test B (*n*[%] missing: 20[3%])	95.63 (44.32)
Stroop Color‐word (*n*[%] missing: 66[10%])	107.40 (28.14)
*Memory*	
Visual association test A (*n*[%] missing: 24[4%])	11.56 (1.02)
RAVLT (5 trials summed) (*n*[%] missing: 37[6%])	39.59 (8.81)
RAVLT delayed recall (*n*[%] missing: 39[6%])	7.92 (3.04)
*Language*	
Fluency animals (*n*[%] missing: 26[4%])	22.23 (5.84)
Visual association test naming (*n*[%] missing: 24[4%])	11.94 (0.34)
**Structural MRI measures**	
Grey matter volume (mL)	609.50 (85.01)
Fazekas score (median, range)	1 (0–3)
Hippocampus (mL)	7.14 (0.94)
**Basic network parameters**	
Network size	7006.75 (666.91)
Degree	1164.20 (124.17)
Connectivity density	16.63 (1.08)
**Higher‐order network parameters**	
Clustering	0.47 (0.02)
Path length	2.02 (0.02)
Betweenness centrality	7120.14 (701.21)
Gamma	1.69 (0.08)
Lambda	1.10 (0.01)
Small world	1.54 (0.06)

Abbreviations: AD, Alzheimer's disease; CSF, cerebrospinal fluid; gamma, normalized clustering; FTD, frontotemporal dementia; lambda, normalized path length; MCI, mild cognitive impairment; MMSE, mini‐mental state examination; SCD, subjective cognitive decline; VaD, vascular dementia. Number of each neuropsychological tests relative to the entire dataset (*n* = 646) are expressed in *n*[%]. ^1^, 29% missing CSF data (*n* = 162 available). Number of subjects (n) abnormal β‐amyloid1–42, Tau (total), were based on 640 and 375 ng L^−1^ cutoffs, respectively (Mulder et al., 2010; Zwan et al., 2016).

### 2.3 MRI acquisition and preprocessing procedures

T1‐weighted structural MRI scans were acquired at baseline using Magnetom Impact 1.0T (*n* = 121) (Siemens, Erlangen, Germany) and SignaHDxt 3.0T (*n* = 106) (General Electric, Milwaukee, WI) scanners using the following sequences: inversion‐recovery prepared fast spoiled gradient recalled sequence (IR‐FSPGR) at 3.0T (176 slices, matrix= 256 × 256, 1 × 0.9 × 0.9 mm^3^, TE = 3 ms, TR = 7.8 ms, TI = 450 ms, flip angle 12°) and magnetization prepared rapid acquisition gradient‐echo (MPRAGE) at 1.0T (168 slices, matrix= 256 × 256, voxel size = 1 × 1 × 1.5 mm^3^, echo time (TE) = 7 ms, repetition time (TR) = 15 ms, inversion time (TI) = 300 ms, flip angle, 15°). A standard circular head coil was used and head motion was restricted using expandable foam cushions. Statistical parametric mapping version 12 (SPM12), operating in MATLAB (r2012) was used to segment images (resliced: 2 × 2 × 2 mm^3^) into grey matter, white matter and cerebrospinal fluid, and to estimate total grey matter volumes in native space. All segmentations were visually checked for segmentations errors and none had to be excluded.

### 2.4 Network parameters

**Figure 1:**
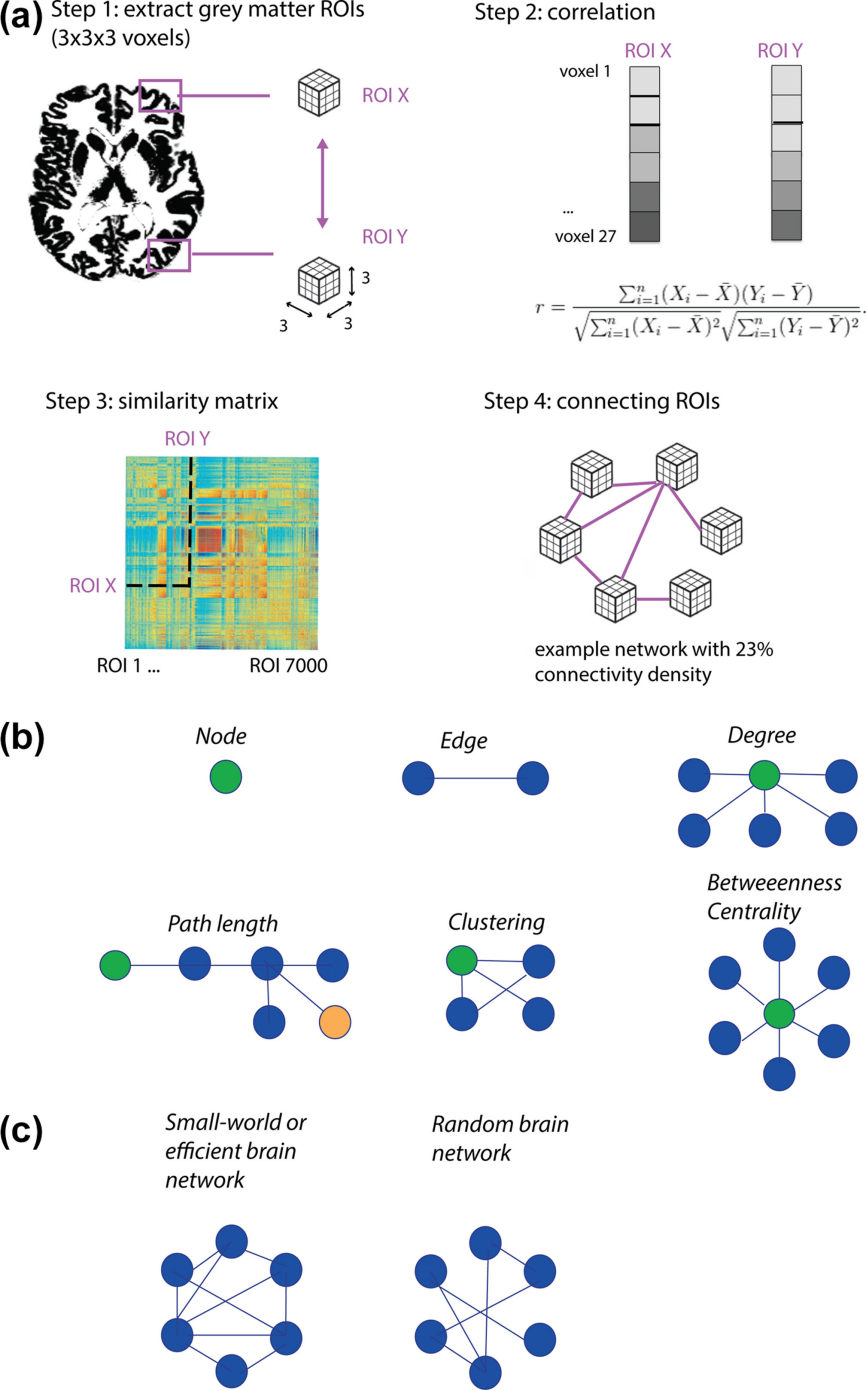
(a) example of single‐subject grey matter network extraction. (Step 1) Grey matter segmentations are divided in regions of interest (ROI) of 3 × 3 × 3 voxels. (Step 2) Connectivity is defined statistical similarity between two ROIs as computed with the Pearson's correlation of grey matter intensity values across corresponding voxels in the ROIs. (Step 3) All similarity values are collected in a similarity matrix. (Step 4) ROIs are connected when their similarity value exceeds a statistical threshold determined with a random permutation method. Here a toy model is shown with an example connectivity density of 23% (i.e., 7 out of 30 possible connections present). (b) Schematic representation of network parameters. A node represents a ROI, and an edge the connection between nodes. The degree is the number of edges of a node, in this example the degree of the green node is 5. Path length is the minimum number of edges between a pair of nodes, in this the path length between the green and orange nodes is 3. Clustering coefficient quantifies to what extent neighbors of a given node are connected, which is 1/3 for the green node as 1 from the 3 possible connections exists. Betweenness centrality is the proportion of paths that run through a node, which is maximal for the green node, and zero for all other nodes. (c) Example of a network with a small‐world organization (left) and with a random organization (right) [Color figure can be viewed at http://wileyonlinelibrary.com]

Single‐subject grey matter networks were extracted from grey matter segmentations (in native space) using a fully automated method implemented in MATLAB (https://github.com/bettytijms/Single_Subject_Grey_Matter_Networks) (see Figure 1 for a schematic overview of methodological steps) (Tijms, Series, Willshaw, & Lawrie, 2012). Briefly, nodes are defined as 3 × 3 × 3 voxels regions in grey matter using an atlas free approach. These nodes keep intact spatial information present in the data, as well as the grey matter density values. Connectivity was then defined by statistical similarity in grey matter structure using Pearson's correlations across the grey matter intensity values of corresponding voxels between any two nodes. All similarity values were collected in a matrix. Nodes were connected using a threshold that ensured that all subjects had a similar chance to include at most 5% spurious connections using a random permutation method (Noble, 2009). Please note that these connections can exist in the absence of an anatomically defined connection. Next, we computed network parameters for each node (local) and/or averaged across nodes (i.e., global). To reduce the number of local tests, we averaged nodal network properties for nodes within each of 90 regions of interest as defined by the automated anatomical labeling (AAL) brain atlas (Tzourio‐Mazoyer et al., 2002) (listed in Supporting Information Table S1). We categorized network measures as being “basic” or “higher‐order” parameters. Basic parameters included the size of a network (i.e., the number of small cortical regions), local and global degree (i.e., the number of edges of a node, which were averaged across nodes of the network to obtain a global estimate), connectivity density (i.e., number of edges relative to network size). Higher‐order network parameters were clustering coefficient (the level of interconnectedness between the neighbors of a node, see Figure 1A for an example), characteristic path length (i.e., the minimum number of edges between a pair of nodes, see Figure 1B for an example) and betweenness centrality (i.e., the proportion of characteristic paths that run through a node, but not start or end at that node). To estimate normalized path length (i.e., lambda) and normalized clustering coefficient (i.e., gamma), we averaged the characteristic path length coefficient and clustering coefficient across the nodes for each network and then divided these properties by those that were averaged across 20 randomized reference networks that had an identical size and degree distribution (Humphries and Gurney, 2008; Maslov and Sneppen, 2002; Watts and Strogatz, 1998). Based on comparisons between AD patients and controls, grey matter networks were considered to be more randomly organized when showing lower gamma and lambda values (Tijms et al., 2013a,b; 2014). We additionally calculated the small‐world property by dividing gamma with lambda coefficients, and a value >1 indicates that a network's topology is different from that of a random graph. Network properties were computed with modified scripts from the Brain Connectivity Toolbox that we (http://www.brain-connectivity-toolbox.net) (Rubinov and Sporns, 2010).

### 2.5 Statistical analyses

Statistical analyses were performed with RStudio (version3.2.5) and Statistical Package for the Social Sciences (SPSS, IBM v22). We used linear mixed models to estimate effects of network measures (predictors) on baseline and longitudinal cognitive performance per cognitive domain (outcome variable). Linear mixed models estimate a coefficient for the longitudinal change based on all data points per cognitive domain available per subject, and handles missing data through maximum likelihood estimation. In the case that effects of basic network parameters (network size, degree, and/or connectivity density) were significant, we added these parameters as additional covariate for models with higher‐order network parameters as predictors, since basic measures can influence higher‐order parameters. Models were run separately for each cognitive domain and each network measure (predictor), including time in years as fixed effect, an interaction term of network measure*time, and subject as random effect. All analyses were adjusted for age, gender, education, total grey matter volume and scanner. Estimates (unstandardized Beta's with standard errors [SE]) were pooled over fifteen imputed data. The false discovery rate (FDR) procedure was used to correct for multiple testing (Benjamini and Yekutieli, 2001). Local network associations were assessed by repeating our analyses for 90 AAL brain regions, additionally adjusted for local grey matter volume, and reported if *p* < 0.05_FDR_.

## 3 RESULTS

Two hundred thirty‐one individuals with SCD (54% male, age: 63 ± 9, MMSE: 28 ± 2) were followed for an average of 3 (SD = 1) years (Table 1). Of 162 subjects with CSF data available at baseline, 40 (25%) had abnormal β‐amyloid 1–42, and 30 (19%) had abnormal tau levels. During the time of study, the majority of subjects (195, 84%) remained clinically stable, whereas 28(12%) developed MCI, 4(2%) developed AD dementia, 2 (1%) frontotemporal dementia and 2 (1%) vascular dementia. During this time period, subjects showed deterioration in language functioning (β = −0.14, SE = 0.05, *p* < 0.05_FDR_). No changes over time were observed in memory (β = 0.00, SE = 0.02), attention (β = −0.01, SE = 0.02) executive functioning (β = −0.03, SE = 0.02) or global cognition (β = −0.42, SE = 0.23) (all *p* > 0.05_FDR_).

There were no associations between basic network parameters and concurrent cognitive performance (all *p* > 0.05_FDR_; Table 2). Linear mixed models showed that smaller network size was associated with worse language over time (*p* < 0.05_FDR_). There were no other associations between basic network parameters and longitudinal cognitive performance (*p* > 0.05_FDR_).

**Table 2:** Higher‐order grey matter network parameters in association with baseline and longitudinal cognitive functioning

Basicparameters	Attention		Memory		Executive function		Language		Global cognition	
	Baseline	Annual change	Baseline	Annual change	Baseline	Annual change	Baseline	Annual change	Baseline	Annualchange
Network size	0.17 ± 0.08*	−0.02 ± 0.02	−0.18 ± 0.10	0.03 ± 0.02	0.11 ± 0.09	0.00 ± 0.02	0.08 ± 0.11	0.12 ± 0.05**	0.07 ± 0.06	0.03 ± 0.02
**Degree**	0.04 ± 0.08	−0.01 ± 0.02	−0.13 ± 0.09	−0.12 ± 0.09	−0.03 ± 0.08	0.02 ± 0.02	0.02 ± 0.10	0.11 ± 0.05*	0.00 ± 0.06	0.03 ± 0.02
**Connectivity density**	−0.07 ± 0.05	0.01 ± 0.02	0.03 ± 0.06	0.02 ± 0.06	0.09 ± 0.06	0.02 ± 0.02	−0.06 ± 0.07	−0.01 ± 0.05	−0.04 ± 0.04	0.01 ± 0.02
**Higher‐order parameters**										
**Clustering**	−0.02 ± 0.06	0.02 ± 0.02	0.04 ± 0.07	0.04 ± 0.02	−0.08 ± 0.05	0.03 ± 0.02	−0.13 ± 0.25	0.04 ± 0.05	−0.03 ± 0.04	0.03 ± 0.02
**Path length**	−0.07 ± 0.05	−0.01 ± 0.02	0.06 ± 0.06	0.03 ± 0.02	0.08 ± 0.05	0.00 ± 0.02	−0.02 ± 0.08	0.11 ± 0.05**	0.03 ± 0.04	0.05 ± 0.02**
**Betweenness centrality**	0.35 ± 0.26	−0.02 ± 0.02	−0.15 ± 0.11	0.04 ± 0.02	0.16 ± 0.09	0.00 ± 0.02	0.05 ± 0.14	0.14 ± 0.05**	0.10 ± 0.07	0.03 ± 0.02
**Gamma**	0.22 ± 0.11	0.01 ± 0.02	0.10 ± 0.08	0.05 ± 0.02*	0.02 ± 0.07	0.02 ± 0.02	0.05 ± 0.09	0.11 ± 0.04**	0.01 ± 0.05	0.06 ± 0.02**
**Lambda**	0.09 ± 0.06	0.02 ± 0.02	0.09 ± 0.07	0.04 ± 0.02	0.04 ± 0.06	0.02 ± 0.02	−0.01 ± 0.07	0.12 ± 0.05**	0.02 ± 0.04	0.06 ± 0.02**
**Small world**	0.21 ± 0.11	0.01 ± 0.02	0.08 ± 0.08	0.05 ± 0.02*	0.11 ± 1.11	0.25 ± 0.32	0.07 ± 0.09	0.11 ± 0.05**	0.01 ± 0.05	0.05 ± 0.02**

Data are presented as beta estimates ± standard error with significance levels *, *p* < 0.05; **, *p* < 0.05 all FDR‐corrected. Additional adjustments per cognitive domain were done if estimates of network size and/or degree were significant at baseline. Attention was additionally corrected for network size, language for size and degree. Estimates are presented from models with age, gender, educational level and total grey matter volume as covariates. Gamma, normalized clustering; lambda, normalized path length.

**Figure 2:**
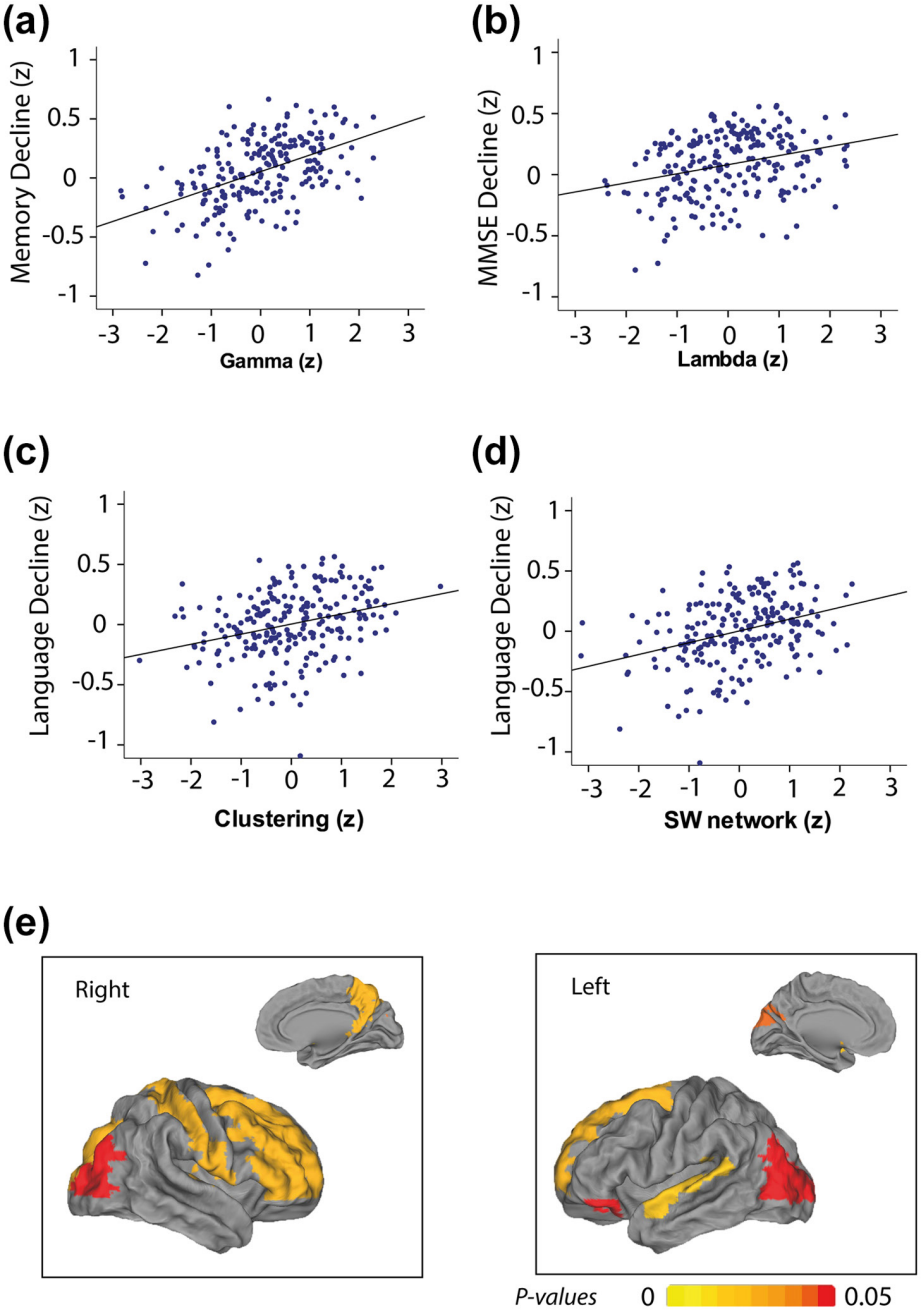
(a) Associations between gamma (i.e., normalized clustering) values and memory changes over time. (b) Associations between lambda (i.e., normalized path length) values and global cognitive changes over time. (c) Associations between clustering values and language changes over time. (d) Associations between small world network values and language changes over time. Predicted changes over time (fixed effects) were obtained with the fitted linear mixed models on the original data. (e) Surface plot of AAL areas where lower path length values were associated with steeper decline of global cognition (*p* < 0.05 FDR‐corrected) [Color figure can be viewed at http://wileyonlinelibrary.com]

There were no associations between higher‐order parameters and baseline cognitive functioning (Table 2). Several associations between higher‐order parameters and cognitive decline were found (Figure 2): Lower baseline values of gamma were associated with a steeper annual decline in language (β = 0.11, SE = 0.04, *p* < 0.05_FDR_) and global cognition (β = 0.06, SE = 0.02, *p* < 0.05_FDR_). Lower lambda values were associated with a steeper decline in global cognition (β = 0.06, SE = 0.02, *p* < 0.05_FDR_) and language (β = 0.15, SE = 0.05, *p* < 0.05_FDR_). Lower baseline values of betweenness centrality and path length were associated with a steeper decline in language functioning (respectively β = 0.14, SE = 0.05; β = 0.11, SE = 0.05; Figure 1E; all *p* < 0.05_FDR_). Lower small world network values were associated with a steeper decline in language (β = 0.11, SE = 0.05, *p* < 0.05_FDR_) and global cognition (β = 0.05, SE = 0.02, *p* < 0.05_FDR_). Repeating analyses when additionally adjusting for hippocampal volumes did not essentially change these results, suggesting that network parameters have additive explanatory values over simpler volumetric measures (Supporting Information Table S2).

We further investigated whether language and global cognitive decline were associated with network alterations in specific anatomical areas. Lower path length values in the left superior frontal, right middle frontal, left inferior frontal, left cuneus, right superior occipital, left middle occipital, left precuneus, right transverse temporal gyri (all *p* < 0.05_FDR_) were associated with faster decline in global cognition (Figure 2E). Local associations between other higher‐order network parameters and language decline did not survive correction for multiple comparisons.

## 4 DISCUSSION

Individuals with SCD who had a more randomly organized grey matter network at baseline showed steeper decline in language and global cognition over time. These associations were independent of hippocampal volumes, suggesting that grey matter network properties can explain variance in cognitive functioning in addition to medial temporal atrophy. Our findings suggest that at very early, preclinical stages, a more randomly organized grey matter network may reflect one of the earliest brain changes associated with subsequent cognitive decline.

In the dementia stages of AD grey matter networks seem to show a more random topology, as indicated by a reduced normalized clustering (i.e., gamma) and/or normalized path length (i.e., lambda) (He, Chen, & Evans, 2008; Pereira et al., 2016; Tijms et al., 2013a; Yao et al., 2010). In the present study we investigated whether a more random grey matter network organization is associated with early cognitive changes in individuals with SCD. Self‐perceived cognitive decline in cognitively normal individuals is associated with a three to six fold increased risk of AD, and could be an early sign of underlying neurodegenerative disease (Geerlings, Jonker, Bouter, Adèr, & Schmand, 1999; Jessen et al., 2010; Schmand et al., 1996). To our knowledge, grey matter networks in relation with subsequent cognitive decline has only been investigated in MCI (Pereira et al., 2016; Yao et al., 2010), and those studies reported higher as well as lower values for gamma in MCI compared to controls. We recently reported, in a partly overlapping sample, that a more randomly organized single‐subject grey matter networks, in particular lower values for clustering and gamma, were associated with increased risk of clinical progression in nondemented amyloid positive individuals (Tijms et al., 2017). In the present study we furthermore showed that lower gamma and lambda values were associated with longitudinal decline in language, which is often impaired in AD dementia (Smits et al., 2015). These findings are in line with a former study showing associations of lower lambda and gamma values with worse memory and language impairment in AD dementia (Tijms et al., 2014). However, not all higher‐order network characteristics in the present study seem to point to AD pathophysiology. For example, we observed associations between betweenness centrality and subsequent decline in cognitive functioning, while in a former study we did not observe any differences in betweenness centrality values between AD patients and controls (Tijms et al., 2013), nor did we observe associations of this network parameter with amyloid pathology in cognitively normal individuals (Tijms et al., 2016, 2013a). Possibly, our present associations between betweenness centrality and language decline might be part of normal aging, or can be due to pathological processes unrelated to AD. In recent studies in cognitively normal individuals, we have demonstrated associations between amyloid abnormalities and path length in fronto‐temporo‐parietal regions (Tijms et al., 2016), and thinner temporal cortex to be related to memory decline and disease progression (Pegueroles et al., 2017; Verfaillie et al., 2017, 2016). We now show that lower path length values in fronto‐temporo‐occipital cortices and precuneus were associated with global cognitive decline. It could be speculated that the earliest path length changes might originate in the precuneus, one of the brain areas involved in the early amyloid deposition (Villeneuve et al., 2015), from which later network alterations may spread to the fronto‐temporo‐occipital cortices. The biological meaning of higher‐order network values is not yet entirely clear, but lower clustering coefficient values might indicate an a‐synchronized deterioration of brain morphology. At the same time, such a‐synchronized patterns of atrophy could potentially lead to more “spurious” correlations between brain areas that previously did not show similarity before, and this might be reflected by lower lambda values. More longitudinal research is required to further investigate potential pathophysiological mechanisms that are associated with these early, preclinical, grey matter network changes.

Among the strengths of our study is the availability of repeated neuropsychological assessment in a unique and relatively large sample of subjects with SCD. Nevertheless, several potential limitations need to be considered. SCD subjects received follow‐up of variable duration that was based on clinical indications. For this reason, it is unclear to which extent our results can be generalized to community‐dwelling individuals with SCD. Second, subjects were scanned at two different scanner systems. Although we corrected our analyses for scanner system, the possibility that this has influenced the results cannot be excluded. Third, since about 30% of our subjects had no CSF available, we cannot exclude the possibility that other pathophysiological events may have influenced these associations. Lastly, in the current study there was no information available about visuo‐spatial functioning, while these are frequently impaired in AD, and found to be related to grey matter networks (Tijms et al., 2014). Future studies are necessary to further elucidate potential associations between grey matter networks and visuo‐spatial functions in cognitively unimpaired individuals.

In sum, we observed that individuals with SCD who had a more randomly organized grey matter network showed faster decline in global cognition and language. These findings suggest that grey matter networks could reflect very subtle structural brain changes that may foreshadow objective cognitive decline.

## Supporting information

Additional Supporting Information may be found online in the supporting information tab for this article.

Supporting Information
