# Supplementary material for: A more randomly organized grey matter network is associated with deteriorating language and global cognition in individuals with subjective cognitive decline
Source: Hum Brain Mapp. 2018 Mar 30;39(8):3143–51. doi: 10.1002/hbm.24065 (PMC6055627; doi:10.1002/hbm.24065)
Supplement: Supplementary file 1 — Supporting Information [file HBM-39-3143-s001.docx]

SUPPLEMENTARY MATERIALS

**Supplementary table S1. Anatomical regions**

| **ROI no.** | **ROI name** | | **ROI no.** | **ROI name** |
| --- | --- | --- | --- | --- |
| 1 | Precentral gyrus | | 24 | Lingual gyrus |
| 2 | Superior frontal gyrus | | 25 | Superior occipital |
| 3 | Superior orbito-frontal gyrus | | 26 | Middle occipital |
| 4 | Middle frontal gyrus | | 27 | Inferior occipital |
| 5 | Middle orbito-frontal gyrus | | 28 | Fusiform |
| 6 | Inferior frontal operculum | | 29 | Postcentral gyrus |
| 7 | Inferior frontal triangularis | | 30 | Superior parietal |
| 8 | Inferior orbito-frontal gyrus | | 31 | Inferior parietal |
| 9 | Rolandic operculum | | 32 | Supramarginal gyrus |
| 10 | Supplementary motor area | | 33 | Angular gyrus |
| 11 | Olfactory gyrus | | 34 | Precuneus |
| 12 | Superior medial frontal gyrus | | 35 | Paracentral lobule |
| 13 | Superior medial orbito-frontal gyrus | | 36 | Caudate |
| 14 | Gyrus Rectus | | 37 | Putamen |
| 15 | Insula |  | 38 | Pallidum |
| 16 | Interior cingulate | | 39 | Thalamus |
| 17 | Middle cingulate | | 40 | Heschl’s gyrus |
| 18 | Posterior cingulate | | 41 | Superior temporal gyrus |
| 19 | Hippocampus | | 42 | Superior temporal pole |
| 20 | Parahippocampal gyrus | | 43 | Middle temporal gyrus |
| 21 | Amygdala | | 44 | Middle temporal pole |
| 22 | Calcarine | | 45 | Inferior temporal gyrus |
| 23 | Cuneus |  |  |  |

Regions were derived based upon the automated anatomical labeling (AAL) brain atlas which consists of 45 ROIs, mirrored in each hemisphere (n=90 ROIs). Abbreviation: ROI, region of interest.

**Supplementary table S2. Grey matter network associations with baseline and longitudinal cognitive functioning with additional adjustment for hippocampus**

| **Basic**  **parameters** | | **Attention** | | **Memory** | | **Executive Function** | | **Language** | | **Global Cognition** | |
| --- | --- | --- | --- | --- | --- | --- | --- | --- | --- | --- | --- |
|  |  | Baseline | Annual change | Baseline | Annual change | Baseline | Annual change | Baseline | Annual change | Baseline | Annual change |
| **Network Size** | | 0.18±0.08* | -0.02±0.02 | 0.19±0.10 | 0.03±0.02 | 0.11± 0.09 | 0.00±0.02 | 0.08±0.11 | 0.12±0.05** | 0.07±0.07 | 0.03±0.02 |
| **Degree** | | 0.05±0.08 | -0.01±0.02 | -0.14±0.09 | -0.04±0.02 | -0.03±0.08 | 0.02±0.02 | 0.02±0.10 | 0.11±0.05* | 0.00±0.06 | 0.03±0.02 |
| **Connectivity Density** | | -0.07±0.05 | 0.01±0.02 | 0.02±0.07 | 0.02±0.02 | 0.09±0.06 | 0.02±0.02 | -0.06±0.07 | -0.01±0.05 | -0.04±0.04 | 0.01±0.02 |
|  |  |  |  |  |  |  |  |  |  |  |  |
| **Higher-order parameters** | | |  |  |  |  |  |  |  |  |  |
| **Clustering** | | -0.02± 0.06 | 0.02±0.02 | 0.04± 0.07 | 0.04±0.02 | -0.08± 0.06 | 0.03±0.02 | -0.13±0.25 | 0.04±0.05 | -0.03±0.04 | 0.03±0.02 |
| **Path Length** | | -0.05±0.07 | -0.01±0.02 | 0.06±0.06 | 0.03±0.02 | 0.08±0.05 | 0.00±0.02 | -0.02± 0.08 | 0.11±0.05** | 0.03±0.04 | 0.05±0.02** |
| **Betweenness Centrality** | | 0.35±0.26 | -0.02±0.02 | -0.17±0.11 | 0.04±0.02 | 0.16± 0.09 | 0.00±0.02 | 0.05± 0.14 | 0.14±0.05** | 0.10±0.07 | 0.03±0.02 |
| **Gamma** | | 0.23±0.11 | 0.01±0.02 | 0.10± 0.08 | 0.05±0.02* | 0.02± 0.07 | 0.02±0.02 | 0.06± 0.09 | 0.12±0.05** | 0.01±0.05 | 0.06±0.02** |
| **Lamdba** | | 0.09±0.06 | 0.02±0.02 | 0.09± 0.07 | 0.05±0.02 | 0.04± 0.06 | 0.02±0.02 | -0.01±0.07 | 0.12±0.05** | 0.02±0.04 | 0.06±0.02** |
| **Small World** | | 0.22±0.11 | 0.01±0.02 | 0.09±0.08 | 0.05±0.02* | 0.11±1.11 | 0.25±0.32 | 0.07±0.09 | 0.11±0.05** | 0.01±0.05 | 0.05±0.02** |

Data are presented as beta estimates ± standard error with significance levels *, p<0.05; **, p<0.05 FDR-corrected. Additional adjustments per cognitive domain were done if estimates of network size and/or degree were significant at baseline. Attention was additionally corrected for network size, language for size and degree. Estimates are presented from models with age, gender, educational level and total grey matter volume as covariates with additional correction for hippocampal volume
